# ID15A at the ESRF – a beamline for high speed *operando* X-ray diffraction, diffraction tomography and total scattering

**DOI:** 10.1107/S1600577519016813

**Published:** 2020-01-28

**Authors:** Gavin B. M. Vaughan, Robert Baker, Raymond Barret, Julien Bonnefoy, Thomas Buslaps, Stefano Checchia, Denis Duran, Francois Fihman, Pierrick Got, Jerôme Kieffer, Simon A. J. Kimber, Keith Martel, Christian Morawe, Denis Mottin, Emanuel Papillon, Sébastien Petitdemange, Antonios Vamvakeros, Jean-Phillipe Vieux, Marco Di Michiel

**Affiliations:** a ESRF – The European Synchrotron, 71 Avenue des Martyrs, 38000 Grenoble, France; b 6Tec, 745 Route de Grenoble, 38260 La Frette, France; c Finden Ltd, Building R71, Rutherford Appleton Laboratory, Harwell, Oxford OX11 0QX, UK

**Keywords:** *operando* diffraction, X-ray diffraction computed tomography, total scattering, time-resolved diffraction

## Abstract

ID15A is a newly refurbished beamline at the ESRF devoted to *operando* and time-resolved diffraction and imaging, total scattering and diffraction computed tomography. The beamline is optimized for rapid alternation between the different techniques during a single *operando* experiment in order to collect complementary data on working systems. The high available energy (up to 120 keV) means that even bulky and highly absorbing systems may be studied. The beamline is equipped with optimized focusing optics and a photon-counting CdTe pixel detector, allowing for both unprecedented data quality at high energy and for very rapid triggered experiments. A large choice of imaging detectors and ancillary probes and sample environments is also available.

## Abstract 2

ID15A is a newly refurbished beamline at the ESRF devoted to *operando* and time-resolved diffraction and imaging, total scattering and diffraction computed tomography.

## 1. Introduction   

A characterization challenge for materials scientists is to bring the conditions of experiments as close as possible to the real conditions under which the systems being studied operate. To do so, the time and length scales characteristic of the properties of interest, on non-optimized samples in terms of crystallinity and morphology, must be probed in an environment as close as possible to that under which the process under study takes place.

We will describe the upgraded ESRF-ID15A beamline which has been designed to exploit a range of techniques recently developed to characterize the atomic-level structure of samples such as amorphous, poorly crystalline or micrometre-scale materials, particularly *in situ* as constituents of heterogeneous devices.

The principal activity on the refurbished ID15A centres around *operando* materials chemistry experiments. A multi-technique approach has been adopted to study heterogeneous, real working systems with a combination of high spatial, temporal and structural resolution. Systems such as batteries, fuel cells and catalytic reactors, disordered systems and glasses are the main research topics but the methods used can be applied to almost any topic in materials chemistry and beyond, with recent experiments having been carried out on thermoelectrics, multiferroics, biomaterials, pharmaceuticals and geological and cultural heritage samples.

The three main techniques available at the new ID15A are X-ray diffraction tomography, time-resolved diffraction including sub-microsecond stroboscopic diffraction and total scattering.

The technique of X-ray diffraction computed tomography (XRD-CT) (Harding *et al.*, 1987 ▸; Kleuker *et al.*, 1998 ▸; Bleuet *et al.*, 2008 ▸; Jacques *et al.*, 2011 ▸) offers much richer information on the chemical and microstructural state of the sample than absorption or phase-contrast tomography, as each tomographically reconstructed volume element of the sample contains not a scalar quantity but an entire scattering pattern. Such scattering contrast is reliant on differences in atomic-level configuration rather than X-ray absorption or refraction, and thus even materials with essentially identical X-ray absorption or refraction can be easily spatially differentiated and characterized (Beale *et al.*, 2014 ▸). Furthermore, implementation of pair distribution function methods allows even amorphous samples to be characterized in this way via pair distribution function computed tomography (PDF-CT) (Jacques *et al.*, 2013 ▸).

In the domain of time-resolved experiments, the acquisition of the recently developed DECTRIS Pilatus3 X CdTe 2M detector with high detection efficiency up to 100 keV has opened up the completely new field of very high time-resolved studies on highly absorbing materials. It allows millisecond time resolution on irreversible systems, and sub-microsecond resolution on reversible phenomena using stroboscopic acquisition modes now applicable to classes of phenomena that previously could only be studied at lower energy (<20 keV) due to the characteristics and low efficiency of the detectors employed.

A third major area on ID15A is total scattering studies, which are experiencing a rapid growth in popularity worldwide (Billinge, 2019 ▸), thanks to the availability of advanced software and improved experimental capabilities. For such experiments, it is crucial to have an extended range of *q*-space available for measurement, making synchrotrons, in particular high-energy synchrotrons, the facilities of choice for such studies. As such, the ESRF is uniquely well placed within Europe, and ID15A in particular has been optimized to carry out such studies.

The beamline supports both a full user program as well as substantial industrial activities, with industrial experiments increasingly employing advanced characterization techniques such as XRD-CT, subsequent to their increased accessibility for all users as implemented on ID15A.

## 2. Technical design   

The refurbished ID15A has been designed to provide full compatibility and optimal performance with the new ESRF Extremely Brilliant Source (EBS), the very low emittance storage ring which was installed in 2019 (Biasci *et al.*, 2014 ▸). Both the optics and experimental stations have been conceived to allow rapid and frequent changes between experimental configurations, *e.g.* between micro-diffraction and imaging modes, typically during single sample measurements. The optical scheme can deliver variable size and band-pass, pre-focused beams to all experimental stations with beam size and energy changes on the minute time scale. Those stations themselves have parallel focusing optics, sample stages and detectors in parallel mounts for quick configuration changes. An overall summary of the beamline characteristics is given in Table 1 ▸.

### 2.1. Beamline layout   

**Figure 1:**
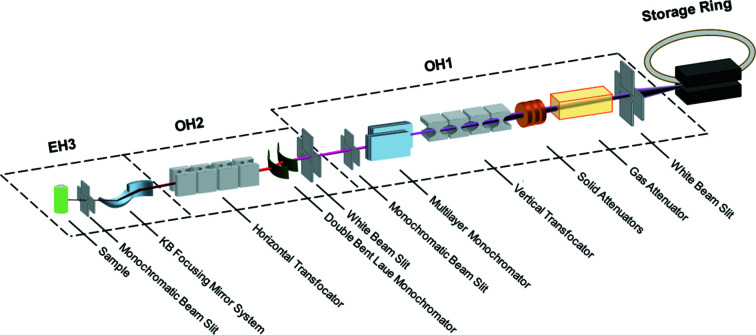
Optical layout of ID15A. In normal operation, the two monochromators are not used simultaneously. EH2 contains no additional optics and is not shown.

The layout of the beamline hutches is shown in Fig. 1 ▸. In order to provide photons to two independent beamlines (ID15A and ID15B), the ID15 straight section is now canted, and will continue to be so with the new lattice (Liuzzo *et al.*, 2018 ▸). The two beamlines operate completely independently apart from sharing a front-end shutter permitting entry to the shared optical hutch OH1, which houses the primary optics of both beamlines.

### 2.2. The X-ray source   

**Figure 2:**
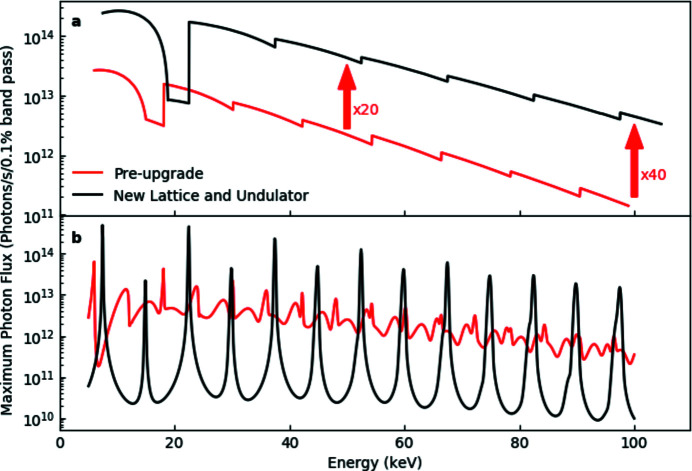
(*a*) Comparison between the photon flux [photons s^−1^ (0.1% band­width)^−1^] of the U22 undulator in the existing lattice (red curve) and of a CPMU18 undulator in the ESRF-EBS lattice (black curve). The calculation is performed for a collecting aperture of 0.3 mm × 0.3 mm at 30 m from the source (primary slit position). (*b*) The calculated undulator spectra with 6 mm gap for the two configurations, illustrating the significant improvement in the shape and peak-to-background ratio with the new lattice.

For initial operation, the refurbished ID15A has operated with a 2 m-long U22 undulator with period λ_u_ = 22 mm and maximum magnetic field of 0.82 T (maximum deflection parameter *K*_max_ = 1.73). In order to fully benefit from the improved performance of the upgraded storage ring, a 1.5 m-long in-vacuum cryogenic permanent-magnet undulator (CPMU18) (Chavanne *et al.*, 2010 ▸) has been constructed, and will be in use from the restart of ESRF operation after the EBS long shutdown in 2020. The undulator period is 18 mm and *K*_max_ = 1.78. The improvement in performance with respect to the prior configuration (Fig. 2 ▸) will be remarkable, with a 20–40× increase in flux at the sample position, as well as significantly sharpened and symmetrized harmonics, particularly at high energy, opening the possibility to use single harmonics with a pinhole as primary source (Vaughan *et al.*, 2011 ▸). The total power from the insertion devices (see Table 1 ▸) will increase only negligibly, due to the shortened length of the new undulator.

A W76 seven-pole wiggler (λ_u_ = 76 mm, *K*_max_ = 13.2) will also be available for very high energy applications up to several hundred keV. The wiggler will offer higher flux and either white or monochromatic unfocused beam above about 150 keV.

### 2.3. Primary optics   

The primary optics are housed in the two optics hutches (OH1 and OH2), and condition the beam size and band-pass as appropriate for a given experiment. Excess heat load, in particular from the unused lower part of the energy spectrum, is eliminated as early as possible to limit thermal strain on downstream optics. The available band-pass and energy range have been extended with respect to those of the former ID15 by deploying two different monochromators. Vertical and horizontal beam size at the sample position have been decoupled via the use of independent vertical and horizontal transfocators equipped with a combination of Be and Al one-dimensional parabolic compound refractive lenses (CRLs).

#### 2.3.1. Slits and attenuators   

New primary slits have been specially designed to cope with the higher heat load of the new lattice and to be effective even at the high energies routinely used on ID15A. The slits are a modified version of the standard ESRF high-power slits (Marion & Zhang, 2004 ▸). The primary modification is the increase of the uncooled W mask thickness to 45 mm, necessary to stop the highest energies.

A subsequent adjustable pressure gas attenuator working with 350 mbar of ultrapure argon serves to effectively eliminate all photon flux below ∼20 keV, without perturbation of the X-ray wavefront. It is crucial to work with ultrapure Ar, as ionized gas impurities [mainly O_2_ and N_2_ at parts per million (p.p.m.) levels] present in less pure argon will rapidly degrade the CVD diamond windows when exposed to the white beam, affecting coherence and ultimately leading to Ar leaks in the vacuum section. A gas purification system is thus used to lower the concentration of impurities from p.p.m. to parts per billion (p.p.b.) levels and consequently reduce the ‘corrosion’ effect by orders of magnitude.

Further attenuation can be achieved, if needed, via a series of three axes of copper-coated diamond filters of the standard ESRF design (Marion *et al.*, 2002 ▸).

#### 2.3.2. Double multilayer monochromator   

A double multilayer monochromator (DMLM) (Morawe, 2019 ▸) is installed in OH1 at 40 m from the source. It provides a monochromatic X-ray beam for XRD and imaging experiments requiring energies below 40 keV, when the use of the Laue–Laue monochromator (LLM) becomes inefficient due to absorption effects. It can also be used to produce a very intense beam for ultra-fast XRD experiments at energies up to 70 keV, when the energy bandwidth is less critical than the flux.

The multilayer mechanical design is based on the standard ESRF water-cooled double mirror/multilayer design (Morawe *et al.*, 2017 ▸) already proven to be extremely stable in terms of vibrations and thermal drifts. The multilayer substrate has two grooves (‘smart cut’) on the substrate sides which keep the slope error within acceptable limits (<0.1 µrad r.m.s.) by simple water cooling when the multilayer is illuminated over the whole length. Detailed calculations of the slope error have been performed to optimize the smart cut dimension *W*_cut_.

**Figure 3:**
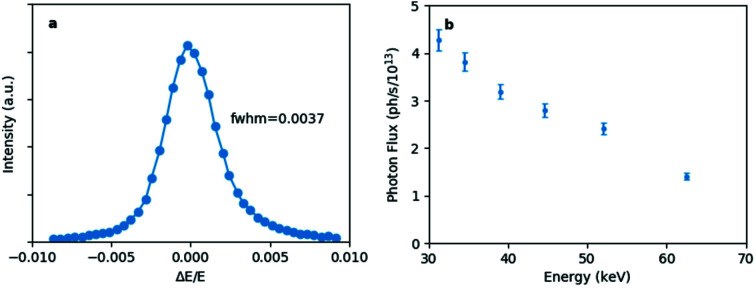
(*a*) Multilayer monochromator energy bandwidth measured at 30 keV using a Si crystal analyser. The bandwidth at FWHM is 0.37%. (*b*) Photon flux density (photons mm^−2^ s^−1^ at 200 mA ring current) provided by the multilayer monochromator at the sample position (unfocused beam).

In order to reduce the energy band-pass, a Ni_93_V_7_/B_4_C layer system was chosen. The *d*-spacing is 1.985 nm at the centre and the substrate length is 300 mm. Both multilayers, working in horizontal scattering geometry, have graded *d*-spacing coatings to compensate for the beam divergence. Due to the transmission of the Ni_93_V_7_ the effective number of active bi-layers could be increased with respect to former instruments from 160 to 500, with a consequent reduction in energy bandwidth from ∼2% to an exceptional measured value of 0.37% [Fig. 3(*a*) ▸], compatible with requirements of many of the ID15A diffraction experiments. The combined reflectivity of the two mirrors is 59% at 20 keV and 86% at 50 keV. The photon flux density of the unfocused beam at the sample position (65 m from the source) provided by the DMLM is shown in Fig. 3(*b*) ▸.

#### 2.3.3. Liquid-nitro­gen-cooled fixed-exit LLM   

The beam­line’s primary monochromator is located at 51 m from the source in OH2. The monochromator has a double-bent-crystal Si (111) Laue geometry of the type previously used at ID15 (Suortti & Tschentscher, 1995 ▸; Tschentscher, 1996 ▸). The monochromatic beam offset is kept constant by moving the first crystal along the beam direction. The horizontal offset and thus the necessary distance between the two crystals has been minimized in order to minimize focal spot broadening from chromatic aberration, which arises from the mismatching polychromatic focal points of the two crystals (Sutter *et al.*, 2008 ▸). The bending assembly has been optimized to give perfect and stable cylindrical bending of the crystals (Honkimäki, 2020 ▸).

The monochromator is liquid-nitro­gen cooled. Heat-load studies by finite-element analysis (FEA) have shown that, even in the most demanding case after the ring and insertion device upgrades, adopting a liquid-nitro­gen cooling system and limiting the absorbed power to ∼150 W, the deformation of the first crystal will be smaller than 0.8 µrad for incoming beam sizes smaller than 2 mm × 2 mm. This corresponds to only 1% of the crystal angular acceptance (∼75 µrad in the energy range 40–100 keV) when bent in Rowland circle geometry. The focus broadening caused by the heat load is also negligible with respect to that caused by chromatic aberration. An asymmetric cut of 36° and a crystal thickness of 3 mm guarantee optimal performances in terms of flux and bandwidth (≤0.3%) in the 40–100 keV energy range.

**Figure 4:**
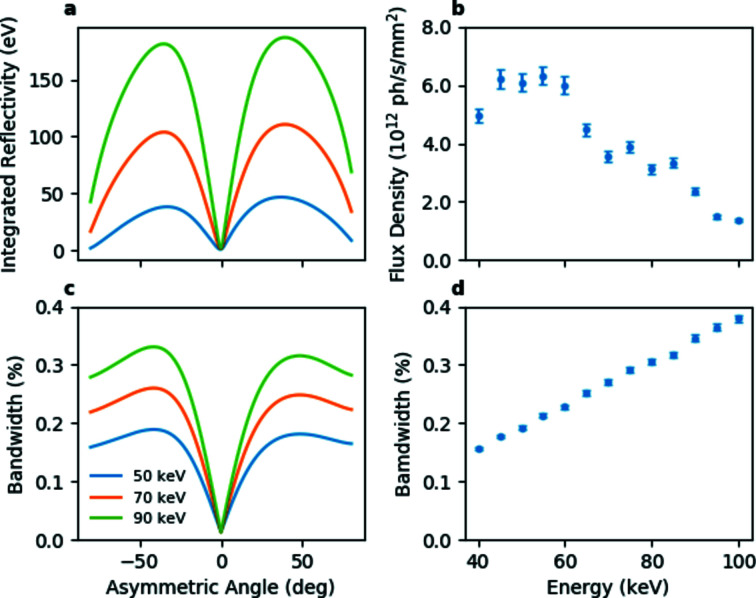
The calculated integrated reflectivity (*a*) and energy bandwidth (*c*) for a double 3 mm-thick Si (111) reflection as a function of the asymmetry angle, and measured photon flux density (*b*) and energy bandwidth (*d*) at the sample position of the LLM (asymmetry angle of 36°) in Rowland circle geometry with an unfocused beam. The apparent irregular behaviour of the photon flux curve is due to the selection of different undulator harmonics.

The calculated integrated reflectivity and bandwidth are shown in Figs. 4(*a*) and 4(*b*) ▸ for the Rowland circle geometry. If desired, the energy bandwidth can be adjusted to the experimental requirements within the range ∼0.01% to ∼1% by modifying the crystal bending radii, although most experiments use optimized (Rowland circle) geometry. Figs. 4(*c*) and 4(*d*) ▸ show the measured photon flux and band-pass at the sample position of an unfocused beam in this geometry. The Si (311) reflection can be used for experiments requiring very high energy X-rays.

The monochromator energy and beam position have also proved to be extremely reproducible; no recalibration of the energy and bending radii or rocking curves are necessary except after shutdowns of the cooling system and ring realignments, nor is it necessary to carry out rocking curves after changing energy. This means that the energy can be routinely changed in a few minutes throughout the entire range, and makes multi-energy experiments as well as the changes between experiments, trivial.

#### 2.3.4. Linear focusing transfocators   

**Figure 5:**
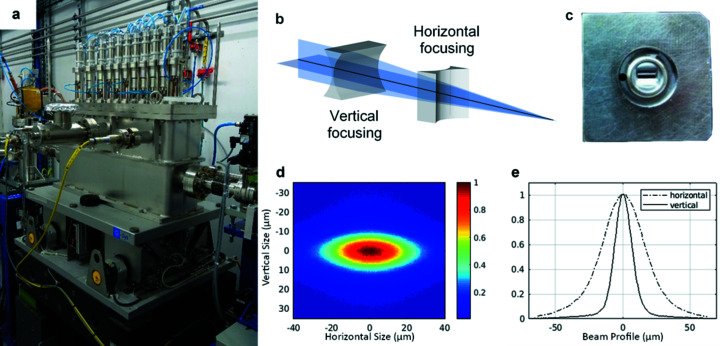
(*a*) The new compact white-beam transfocator vessel. (*b*) Linearly focusing transfocator concept. (*c*) Linear CRL. (*d*) Focal spot size at 50 keV recorded by a high-resolution X-ray imaging camera (pixel size 0.71 µm). (*e*) Measured vertical (solid line) and horizontal (dashed line) beam profiles.

White-beam two-dimensionally focusing transfocators (Snigirev *et al.*, 2009 ▸; Rossat *et al.*, 2010 ▸; Vaughan *et al.*, 2011 ▸) based on CRLs (Snigirev *et al.*, 1996 ▸) are commonly used at synchrotron beamlines as beam condensers or as focusing optics when a focal spot of the order of tens of micrometres is required. Since the synchrotron X-ray horizontal source size is much bigger than the vertical source size the focal spot produced by such transfocators is highly asymmetric. To counteract this phenomenon, at the ID15 complex, for the first time, two crossed linearly focusing white beam transfocators [Fig. 5(*a*) ▸] have been installed which enable independent tuning of the vertical and horizontal focusing to match experimental requirements [Fig. 5(*b*) ▸].

Both transfocators contain sets of Be and Al cylindrical parabolic CRLs with a radius of 100 µm and a geometrical aperture of 630 µm produced by RXOPTICS [Fig. 5(*c*) ▸]. The vertically focusing transfocator (TF1) is located in OH1 at 32 m from the source; it has a source demagnification factor of ∼1. The horizontally focusing transfocator (TF2) is located in OH2 at 53 m from the source, after the LLM; it has a source demagnification factor of 4.4. This choice has been made for several reasons.

By positioning TF2 after the monochromator, the effective broadening of the horizontal source caused by the crystal thickness of the LLM working in horizontal scattering geometry is minimized. This also enables the LLM to work in fixed horizontal Rowland circle geometry, while not affecting the horizontal divergence until after the monochromator.

**Figure 6:**
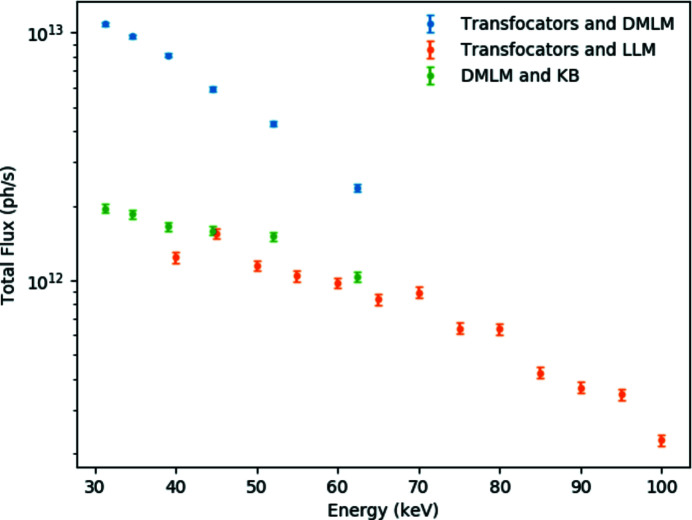
Total flux focused on the sample by the white-beam transfocators with the LLM and DMLM monochromators and with the combination of the DMLM and the KB mirror system. Note that the band-pass and beam size differ in the three cases.

TF1 and TF2 can independently adjust the vertical and horizontal beam size at the sample position in EH3 65 m from the source, where X-ray beams up to 105 keV can be fully focused. The smallest focal spot size achievable with the LLM is 38 µm × 16 µm FWHM (H × V) [Figs. 5(*d*) and 5(*e*) ▸]. A slightly smaller spot (35 µm × 15 µm) has been achieved with the DMLM, most likely as, being in the Bragg configuration, it does not suffer from the effective source broadening due to crystal thickness in the Laue configuration. Following the EBS upgrade, the smallest horizontal focal spot size will be reduced by a factor of ∼2, thus becoming almost equivalent to the vertical size. The measured photon flux on the sample with LLM and DMLM is shown in Fig. 6 ▸.

Considering the improved shape and peak-to-background ratio of undulator harmonics expected with the new storage ring [Fig. 2(*b*) ▸], it is anticipated that for some diffraction experiments, particularly on poorly crystalline materials, a very high flux configuration with a bandwidth of ∼0.6% will be achievable by using a combination of a secondary source produced by the transfocator(s) and a slit to select a single harmonic. Such an arrangement was proposed and validated previously (Vaughan *et al.*, 2011 ▸), and has subsequently been used for some imaging applications, but was of limited interest for diffraction applications due to the shape of undulator harmonics in current storage rings.

### 2.4. Experimental hutch for materials chemistry   

The principle experimental methods employed at ID15A are diffraction from polycrystalline and nanocrystalline materials, and total scattering from these and amorphous samples. Major activities are three-dimensional XRD/PDF tomography of these samples and very fast time-resolved studies, either stroboscopic or single-shot. Absorption tomography is routinely carried out as a complementary technique during scattering experiments, and small-angle scattering (SAXS) is also available. Many experiments rely on a combination of diffraction and imaging, often under *operando* conditions, which entails a constant switch during the experiment between imaging and diffraction modes.

This switch is achieved by the modular conception of the beamline optics and detection. Focusing devices and detectors can be reproducibly moved in and out of the beam on the minute time scale in order to switch between large-beam, high-resolution detection for imaging and small-beam, high-efficiency and dynamic-range detection for diffraction. In EH3, this is done by the permanent mounting of three imaging, one diffraction and one SAXS detectors. Gantries are used to allow quick alignment of instruments, thus minimizing setup time. Parameters of the different configurations are given in Table 2 ▸.

Although absorption from air is negligible at the working energies of ID15A, the incident beam path is kept under vacuum other than a very short distance before the detector to avoid parasitic scattering. Several pairs of slits and collimators are used to clean the beam, and the beamstops used are made of compound materials in order to avoid both fluorescence and low absorption when working near absorption edges. For SAXS measurements, an additional evacuated flight tube is placed between the sample and the detector. The SAXS beamstop is within this flight tube.

**Figure 7:**
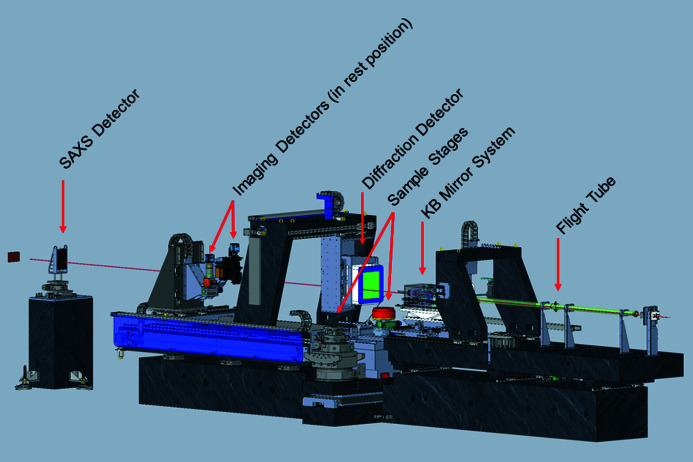
Schematic of the layout of EH3, showing the detector position for diffraction measurements.

A large number of sample environments have been developed and prepared for easy implementation. Another important aspect of fully characterizing materials during chemical changes is to be able to study the samples via a bouquet of complementary probes. In addition to the ubiquitous XRD and X-ray imaging techniques employed at ID15A, many experiments utilize complementary probes which are built into the experimental possibilities at ID15A. These complementary probes include diffuse reflectance infra-red spectroscopy (DRIFTS), mass spectroscopy (MS) and X-ray fluorescence (XRF). A global view of EH3 is shown in Fig. 7 ▸.

The available X-ray energy range in EH3 is 20–140 keV, with much higher energies possible with relatively simple optical realignment. Full-efficiency focusing is possible up to 105 keV. However, because of the declining flux with increasing energy and the trade-off between absorption and Compton cross sections, it is rarely useful to go above ∼120 keV for typical applications in materials chemistry. The beam size can be adjusted from 0.3 µm × 0.6 µm to 6 mm × 8 mm using a variety of optical configurations including using the transfocators to defocus the beam at the sample position.

#### 2.4.1. Secondary optics   

When monochromatic focused beams smaller than those provided by the white-beam transfocators are required, two types of secondary optics are used: two-dimensional focusing CRLs or Kirkpatrick–Baez (KB) mirrors. Both systems are used in combination with the DMLM or the LLM. The advantage of CRL focusing is the rapid alignment or realignment procedure as they do not modify the beam direction. Their displacement of the beam is proportional to their own displacement, and thus the beam position can be very accurate and reproducible when they are inserted and removed. They are preferred when setup time is limited and/or a high degree of automation is required, for example in experiments combining phase/absorption imaging with micro-focusing XRD. The advantages of the KB system are the smaller beam size and the much higher flux provided when beams of the order of a micrometre or smaller are required; this is due to the higher numerical aperture (NA) and reduced attenuation compared with CRLs. For these reasons the KB system is preferred when high spatial and/or time resolution are required, *i.e.* for XRD-CT experiments. Moreover, a more symmetric spot size can be obtained.

Currently, purpose-mounted CRLs in EH3 can be used to produce a horizontal beam size of the order of 3–20 µm at the sample position, with a horizontal to vertical aspect ratio which will be reduced to 3.5 following the EBS upgrade.

**Figure 8:**
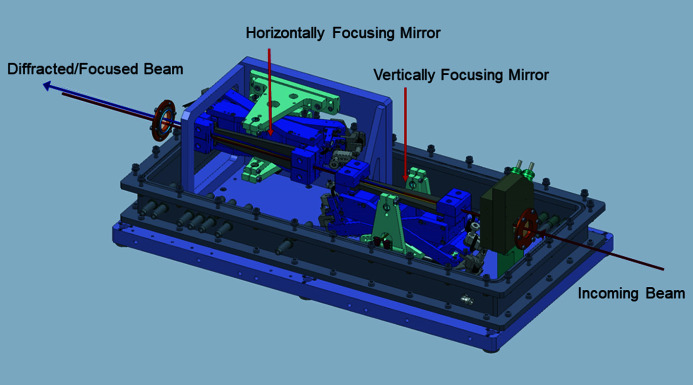
Schematic of the KB system.

The KB system (Fig. 8 ▸) is installed on the optical granite table before the sample position. The distance between the end of the system and the sample position, 0.20 m, is sufficient to insert a wide variety of sample environments without particular spatial constraints. The KB mechanics is a standard ESRF design (Dabin *et al.*, 2002 ▸; Morawe, 2019 ▸). The elliptically bent, trapezoidal-shaped KB mirrors are coated with laterally graded multilayers, with 200 W/B_4_C layers, with a central period of 2.265 nm, giving a bandwidth of ∼1.5% and reflectivity varying from ∼42% at 20 keV to ∼80% at 65 keV. As seen in Fig. 6 ▸, when used in combination with DMLM a focused beam flux of ∼10^12^ photons s^−1^ can be achieved.

**Figure 9:**
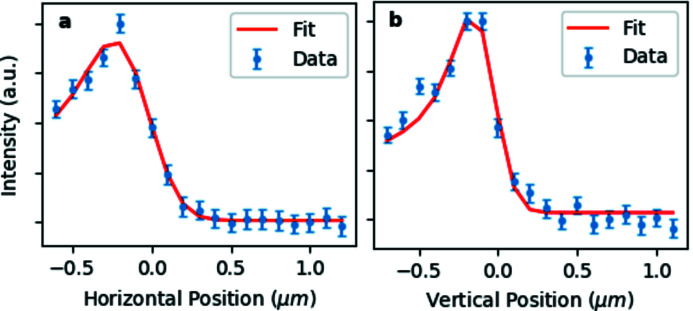
Derivative of the beam intensity (dots) obtained at 60 keV by scanning the KB focal spot with a 200 µm-diameter Au wire (*a*) horizontally and (*b*) vertically. The derivative of the convolution between a Gaussian function of unknown width (KB focal spot) and the wire transmission function is used as a model for the fit (continuous line). The vertical and horizontal FWHM are 0.3 µm and 0.6 µm, respectively.

**Figure 10:**
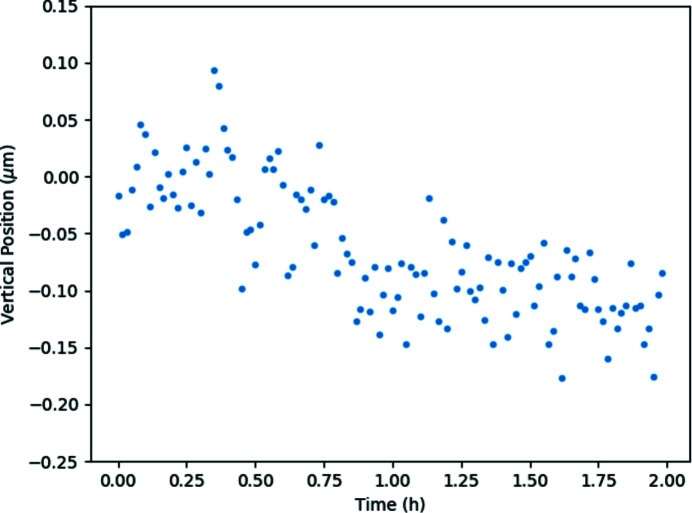
KB beam drift measured over 2 h using a high-resolution imaging system and centre-of-mass method. Measurements were performed every minute, with an exposure time of 0.5 ms.

Contrary to the CRLs the source demagnification factor is fixed, corresponding to 105 for the vertically focusing mirror and 185 for the horizontally focusing mirror. The smallest focal spot achievable prior to the ring upgrade was 0.3 µm × 0.6 µm (V × H) FWHM (Fig. 9 ▸), with the vertical dimension limited by the mirror slope errors and the horizontal dimension by the source size. Larger focal spots can be best achieved by simply moving the sample out of focus. The system has been optimized for use with ESRF-EBS, and will provide a symmetric spot of 0.3 µm × 0.3 µm (V × H) FWHM without any changes in the system. When the KB system is combined with the DMLM the photon flux on the sample is ≥10^12^ photons s^−1^ over the whole spectrum which allows extremely fast measurements, on a time scale unachievable with the old ID15A beamline. Furthermore, the KB system is extremely stable, a necessity for carrying out spatially resolved *operando* measurements on realistic time scales. Fig. 10 ▸ shows measurements of the KB focused beam position relative to the sample positon carried out over two hours with a high-resolution imaging camera system and sub-millisecond exposure time using the centre of mass method; the spread in the data points represents high-frequency vibrations in the detector mount. Similar results (with lower time resolution) are obtained using a beam position monitor. The long-term drifts, however, are almost inconsequential and easily correctable, particularly after thermal equilibrium is reached

#### 2.4.2. Sample stages   

Two sample stages have been designed for EH3, one for experiments requiring heavy and/or bulky sample environments and one for high spatial and temporal resolution experiments (Fig. 7 ▸). The two sample stages are permanently mounted on a granite translation stage orthogonal to the beam which allows rapid and reproducible changes between the two configurations.

The heavy-duty sample stage accepts a maximum load of 150 kg. It can carry a large variety of beamline and user setups and sample environments such as the DRIFTS spectrometer and associated catalytic cell and mass spectrometer, ball mills, compression and torsion rigs, large furnaces, *etc*.

The high-precision sample stage is used for multidimensional *operando* experiments and in general for all experiments requiring a high positioning accuracy and/or high-speed rotation. The system is also equipped with a rotating electrical connection (slip-ring) to pass currents and other electrical signals. This eliminates the necessity to rewind the rotation stage, and allows continuous sample rotation during *operando* computed tomography (CT) (*i.e.* battery experiments), thus reducing the data acquisition time. The current stage has 100 nm sample positioning accuracy, 200 r.p.m. rotation speed and 40 kg maximum load. If necessary, additional translation and/or rotation stages for positioning samples may be placed on top of the permanent stages.

#### 2.4.3. X-ray detectors   

A variety of detectors are permanently mounted in parallel in EH3, in order to perform near simultaneous wide-/small-angle scattering and imaging experiments. Wide-angle scattering is carried out with a DECTRIS Pilatus3 X CdTe 2M detector, a single-photon-counting fast area detector designed for high-energy X-ray measurements. The Pilatus3 X detector data quality and data acquisition speed are far superior to that of the flat-panel detectors used by most high-energy diffraction beamlines. The main detector parameters are listed in Table 3 ▸, compared with the Perkin Elmer flat-panel detector previously used on ID15A.

The single-photon-counting feature eliminates the detector electronic noise, allowing the measurement of much weaker signals or signal differences (smaller samples, concentrations, *etc.*). As the detector is a true counting detector, data quality can be more easily quantified statistically and counting schemes optimized. Furthermore, with a 20-bit dynamic range, very intense and very weak signals can be measured simultaneously, in order to identify weak phases or subtle features in the diffractogram, or to measure weak signals against very high backgrounds such as from sample environments.

Electronic gating (with minimum exposure time of 200 ns) allows high-energy stroboscopic X-ray diffraction with area detectors to be performed for the first time. The counting linearity up to 5 × 10^6^ photon s^−1^ pixel^−1^ allows accurate measurements with a very high photon flux during, for example, stroboscopic experiments (Schultheiß *et al.*, 2018 ▸; Liu *et al.*, 2018 ▸).

The adjustable energy threshold eliminates most of the fluorescence signal from the sample and sample environment, which can be highly detrimental to many measurements, particularly total scattering studies, where most of the signal at high-*q* often comes from sample fluorescence.

**Figure 11:**
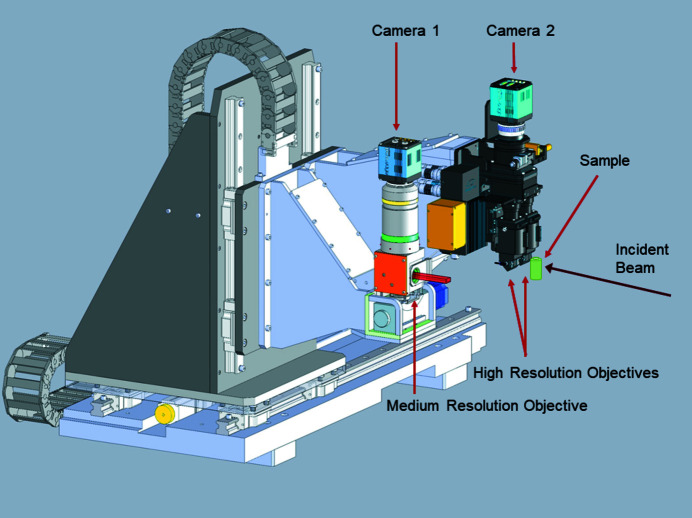
The high- and the medium/low-resolution imaging detectors mounted in EH3 at ID15A.

Two detector systems especially designed for high-energy X-ray fast imaging are permanently installed in EH3 (Fig. 11 ▸). These systems are used in combination with the XRD detectors to obtain information about the sample microstructure evolution, mainly via tomographic scans in absorption or phase propagation mode.

The X-ray detection scheme is indirect: X-rays are absorbed by a scintillator screen and converted into visible-light photons which are collected by a magnifying objective and focused onto a CMOS camera. Different spatial resolutions and fields of view are obtained by selecting scintillator thickness and objective magnification.

Both imaging detector systems use low-noise high-speed PCO edge scientific CMOS cameras. The first has 1× or 2× large aperture magnifying optics and is used for large-field-of-view low/medium-spatial-resolution imaging. Exposure times as low as half a millisecond can be used even with monochromatic beam radiation.

The second imaging detector system, with visible-light optics manufactured by OptiquePeter, is equipped with a motorized double objective-scintillator system for high-spatial-resolution imaging which enables a fast change of magnification and scintillator. Any combination of two 5×, 10× or 20× magnifications can be mounted at a given time and swapped between in seconds, making it feasible to carry out multiple-resolution *in situ* studies. Typical exposure times are tens of milliseconds in monochromatic beam and a few milliseconds with the pink beam coming from a single undulator harmonic, depending on the X-ray energy. The visible-light optics are compatible with this white-beam operation due to the presence of the 45° mirror between the scintillator screen and the long-working-distance objectives which are furthermore protected by Pb-glass lenses.

Both detectors are mounted on the same granite table as the Pilatus WAXS detector. The distance to the sample can be adjusted from 0 to 2 m to work in absorption or phase propagation mode, and imaging can be carried out over the entire energy range of the beamline. The main characteristics of the X-ray detector systems are summarized in Table 4 ▸.

EH3 is also equipped with a Hitachi Vortex-EM silicon drift X-ray fluorescence detector. The detector is combined with a fast Mercury/XIA readout electronics allowing exposure times of a few milliseconds. The Vortex detector is used to perform simultaneous fluorescence mapping and XRD mapping or fluorescence CT and XRD-CT.

In catalysis and energy sector experiments the combination of the two techniques is extremely useful, fluorescence providing elemental concentration and XRD structural information. Due to the thick sensor, the detector can be used to measure fluorescence lines up to 80 keV, and to detect low-energy lines excited with even such high-energy beams, giving access to the *K*-edges of rows 4 to 7 of the periodic table.

In high-energy micro-diffraction experiments the detector is also utilized for precise sample alignment and to identify easily the region of interest for XRD measurements. Moreover, the fluorescence scans are performed in the same condition as the XRD measurements, *i.e.* using an identical incident X-ray beam, and thus do not require the removal of the focusing optics (KB or CRLs), as is necessary for absorption- or phase-contrast imaging. This also means that the positions on the fluorescence map are strictly identical to those used for subsequent/alternating X-ray microdiffraction, facilitating easy comparison.

SAXS is ideally suited for the investigation of nano­structured/nanocomposite functional materials. The combination of SAXS with wide-angle X-ray scattering (WAXS) measurements can provide both structural and morphological information, which is extremely valuable for a deeper understanding of the parameters which govern the formation and the behaviour of different length scale structures present in functional materials. This is notably true for catalysts and modern battery electrodes where nanoparticles are omnipresent.

ID15A is equipped with a single-photon-counting Maxipix CdTe detector (Ponchut *et al.*, 2011 ▸) for time-resolved high-energy SAXS. Measurements over a *q*-range from 0.01 to 1 nm^−1^ are possible at 30 keV, and the CdTe sensor ensures that much higher incident energies can also be used. The maximum frame rate is 350 Hz, making it particularly well suited for time-resolved experiments and SAXS-CT. In addition to the standard acquisition mode, the detector features, similar to the Pilatus3 detector, there is an electronic gating option with a minimum exposure time of 100 ns. This opens new possibilities for high-energy time-resolved SAXS experiments on the sub-microsecond time scale using a stroboscopic approach as already demonstrated with the Pilatus detector. The main detector parameters are listed in Table 5 ▸.

### 2.5. Experimental Hutch 2   

ID15A retains the capability to use very high energy beams (up to 700 keV) to carry out experiments needing these unique conditions, such as strain measurements in angle- or energy-dispersive mode of very highly absorbing materials, high-speed high-energy pink-beam imaging, and more exotic experiments such as the characterization of optics and detectors for X-ray astronomy. These experiments can be carried out in Experimental Hutch 2, which will be described in a future publication (Buslaps, 2020 ▸).

## 3. Experimental infrastructure   

### 3.1. Ancillary probes   

The use of ancillary probes to complement the data acquired in X-ray scattering or imaging has become a crucial part of experimental methodology. At ID15A, a Hiden QGA Quadrupole UHV mass spectrometer can be used for real-time quantitative gas and vapour analysis during chemical reactions. The system is configured for sampling at gas pressures up to 30 bar. The mass range is 200 atomic mass units. It can be used with a Faraday Cup detector (detection limit 10^−11^ mbar) or with a Secondary Electron Multiplier for higher sensitivity (detection limit 10^−13^ mbar or 5 p.p.b.). The maximum measurement speed for a single mass is 1 ms.

**Figure 12:**
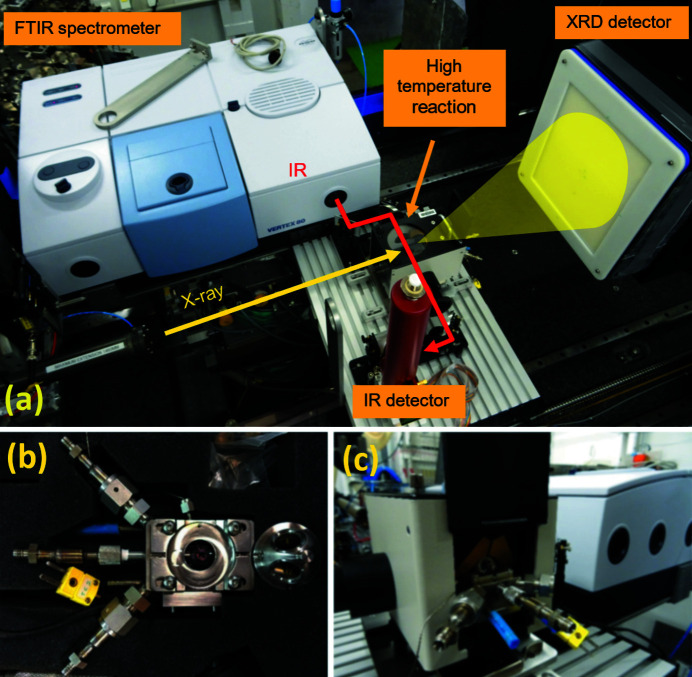
(*a*) Setup for combined FTIR and XRD measurements. (*b*) The Harrick HVC high-temperature high-pressure cell for combined DRIFTS/XRD measurements. (*c*) The cell and the DRIFTS optics.

A Bruker Vertex 80 infrared spectrometer is also available for use during *operando* experiments of chemical reactions. Coupled to a fast, nitro­gen-cooled detector (LN-MCT) it is used for *in situ* experiments where the sample is probed simultaneously by X-rays and IR [Fig. 12(*a*) ▸]. The trigger in/out capability allows synchronization of the collection of IR spectra and X-ray diffraction patterns. The spectrometer can probe the near-IR and middle-IR range (8000–350 cm^−1^) with a resolution up to 0.2 cm^−1^. Coarser resolutions can be used to increase scan speed up to 100 spectra per second (at 16 cm^−1^), and an ultimate time resolution of 5 ns can be achieved in the stroboscopic mode.

In this mode, reversible processes can be tracked by the equivalent technique as with the Pilatus detector for stroboscopic mode diffraction, *i.e.* by triggering the process repeatedly to cover the whole IR spectral range at successive process times. With the advent of the EBS source, when the feasible collection times for X-ray diffraction will further decrease, ultrafast combined Fourier-transform infrared spectroscopy (FTIR)/XRD experiments will be possible, in which the Pilatus detector and the Vertex spectrometer will be able to reveal chemical processes at frequencies up to 4 MHz.

The mass spectrometer and FTIR system can be used simultaneously during, for example, catalysis reactions, giving the possibility to collect simultaneous XRD/IR/MS data on the millisecond time scale. An example of such simultaneous data has been given by Newton *et al.* (2010 ▸).

### 3.2. Sample environments   

A number of different devices are available to control the sample environment during *operando* experiments. Table 6 ▸ summarizes the characteristics of the temperature control devices currently available at ID15A. The commands needed to read and modify their parameters are integrated into the rest of the experiment control system. They provide a variable temperature range, resolution and sample geometry. In general, the more open the geometry, the less accurate the sample temperature, but the more convenient for, for example, multi-probe, XRD-CT or total scattering experiments.

A gas delivery system is permanently mounted on the beamline, allowing experiments requiring rapidly changing gas environments, in order to mimic real working conditions in chemical reactors. Gases are supplied through five permanently installed gas lines (200 bar, three lines for inert gases, one for H_2_-like and one for CO-like gases) and/or temporary lines installed for specific user experiments. There are six programmable mass flow controllers with four 200 bar inputs and two 40 bar inputs. Three back-pressure controllers can adjust the pressure up to 200 bar in the sample cell while keeping the gas flow constant (up to 100 ml min^−1^). Three electrovalves with their control integrated into the beamline control system allow near-instantaneous switching between four different atmospheres. As the system is permanently mounted and certified, safety issues with dangerous gases are minimized, facilitating a greater flexibility in the experiments which can be performed.

A high-temperature high-gas-pressure reaction chamber [Fig. 12(*b*) ▸] is available for combined IR/MS/X-ray experiments [Fig. 12(*c*) ▸]. It is a modified version of the Harrick HVC cell which allows measuring catalytic systems under reaction conditions (Beyer *et al.*, 2014 ▸). Powder samples can be heated from room temperature up to 600°C under gas pressure (up to 10 bar) and constant flow. FTIR spectra are collected in DRIFTS geometry while the same sample is probed by X-rays in transmission. Simultaneously the mass spectra of the reaction products can be collected. The 2θ range accessible through the cell dome opening makes it possible to collect even PDF data using 80–110 keV X-rays (Beyer *et al.*, 2014 ▸).

A Retsch MM400 mixer mill is available for *in situ* studies of mechano-chemical reactions. This field has grown rapidly in recent years to become a major research area on ID15A (Friščić *et al.*, 2012 ▸; Halasz *et al.*, 2013 ▸).

This setup enables the study of reaction kinetics, intermediates and mechanisms of chemical reactions under milling conditions. Wet, dry and controlled-atmosphere grinding are possible using sealed plexiglass reaction vessels. The mixer has been modified for X-ray measurements, allowing the X-rays to pass the mixer body to reach the oscillating reaction vessel. The maximum oscillation frequency is 30 Hz.

### 3.3. Data acquisition and data processing   

In order to take advantage of all of the technical advances described so far in this chapter, it was necessary to, in parallel, optimize the data acquisition strategy in order to achieve the time resolution made possible by the counting statistics. Methods to collect XRD-CT data during the continuous movement of the translation and the rotation axes in order to minimize dead-time associated with rewinding have already been implemented, and successfully applied in *operando* experiments (Liu *et al.*, 2019 ▸; Matras *et al.*, 2019 ▸). These procedures and planned improvements are discussed in more detail in another article (Di Michiel, 2020 ▸).

The data saving and reduction architecture has also been conceived to deal with the consequent high data rates associated with both absorption/phase propagation imaging (typically 1 GB s^−1^) and XRD-CT (up to 500 MB s^−1^).

There is thus a clear need to reduce the data on the time scale of the experiment. For the majority of experiments on ID15A, the powder diffraction data can typically be azimuthally integrated, leading to a 10^3^-fold compression in data size, thus bringing the volume of a full data set from terabytes to gigabytes prior to reconstruction. In order to reduce the data robustly and rapidly, the PyFAI library (Ashiotis *et al.*, 2015 ▸) was extended to run in parallel on a local GPU with the necessary basic filters (Kieffer *et al.*, 2018 ▸). The reduction itself (the integration of one image takes ∼2 ms, with currently ∼8 ms needed to transfer data from the RAM to the GPU) is on the same time scale as the acquisition.

The automated data reduction must be carried out precisely, in order to avoid subsequent re-evaluation. All experimental factors must be taken into account: geometrical detector corrections for intensity and position, detector intensity response and distortion, and careful masking of the image to account for pixels with non-linear or non-Poissonian behaviours (hot pixels, pixels in gaps or inter-wafer bonds, and pixels near such areas). Robust methods have been implemented to furthermore reject outliers based on median or trimmed-mean filters (Vamvakeros *et al.*, 2015 ▸) in order to eliminate spurious signals not automatically detected (cosmic rays, thorium decays, uncharacterized bad pixels, weak parasitic scattering, *etc*.). This procedure is particularly critical when collecting total scattering data where very small artefacts can cause significant degradation of the resultant *G*(*r*).

In some cases, more exotic filters are necessary, for example filtering in Euler space to remove partially measured features, or further types of signal filters to remove signals from, for example, single-crystal minority phases.

Wrapper scripts have then been implemented to apply other corrections, archive metadata, apply optional post-processing [rebinning in *q* and/or position space, conversion to *S*(*q*), *F*(*q*) and/or *G*(*r*), masking via multiple criteria, absorption corrections for XRD-CT data based on absorption CT sinograms, *etc*.], and to write the rebinned/post-processed data into efficient and self-descriptive hdf5 files for subsequent analysis. In the case of absorption and XRD-CT experiments, the tomographic reconstructions can be launched in parallel with the data acquisition and reduction. This functionality will become even more convenient and transparent following the migration of the ESRF to a Python-based instrument control and data acquisition system.

## 4. Results   

Examples of recent results using the various techniques employed on ID15 are given in this section.

### 4.1. High-resolution time-resolved XRD-CT   

**Figure 13:**
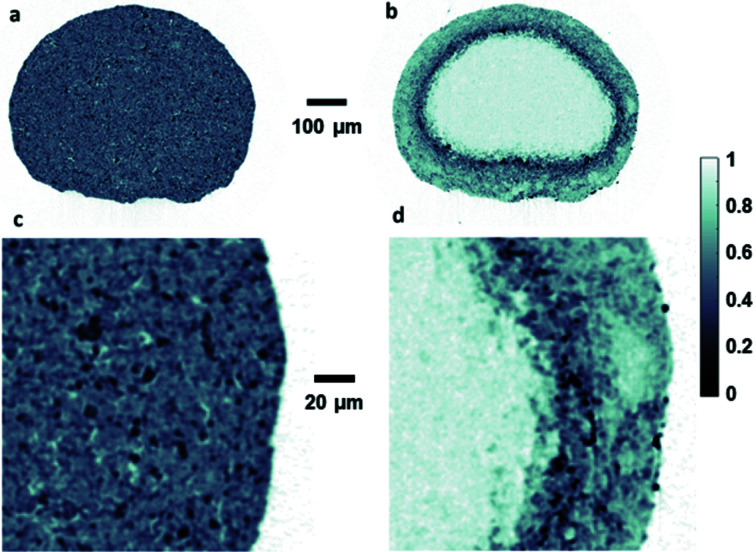
Maps of Al_2_O_3_ (*a*, *c*) and ZrO_2_ phases (*b*, *d*) of a catalyst particle acquired at 50 keV at 1 µm spatial resolution.

The small and stable beam size achieved has allowed XRD-CT experiments to be performed with unprecedented spatial and time resolution, even under *operando* conditions. Fig. 13 ▸ shows phase specific maps from a catalyst particle (diameter ∼600 µm) measured with 1 µm spatial resolution during a recent experiment on the catalytic partial oxidation of methane, a promising alternative method to produce synthesis gas (Vamvakeros *et al.*, 2018 ▸). A total of 480000 diffractograms with 10 ms exposure time each were collected in 1.5 h by the Pilatus3 X CdTd 2M detector working at 50 keV.

### 4.2. Very high energy stroboscopic X-ray diffraction   

**Figure 14:**
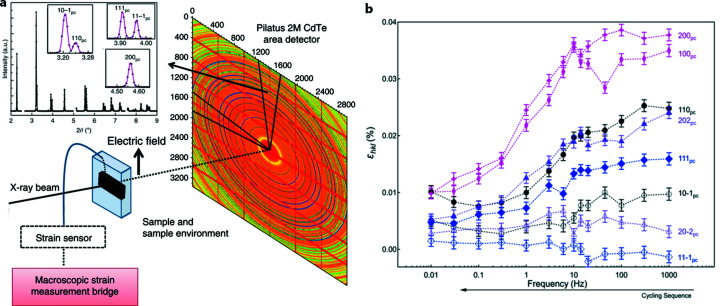
(*a*) Schematic representation of the setup used to measure time-resolved polarization, strain and structural changes simultaneously, with examples of fits to different *q* and angular regions of the azimuthally integrated diffraction patterns. (*b*) Lattice strain ɛ_*hkl*_, from diffraction peak shifts for individual reflections with the scattering vector parallel to the electric field vector, calculated as a function of unipolar field cycling frequency. Adapted from Liu *et al.* (2018 ▸).

Stroboscopic diffraction has been carried out with low-energy X-rays, principally due to the characteristics of the detectors used. With the development of the Dectris CdTe pixel detector, it has become possible to port those experiments to high energy and thus broaden the classes of systems studied. An example of such an experiment is shown in Fig. 14 ▸, in which domain switching in a BiFeO_3_ sample was measured with 10 µs time resolution (Liu *et al.*, 2018 ▸).

### 4.3. Total scattering studies   

**Figure 15:**
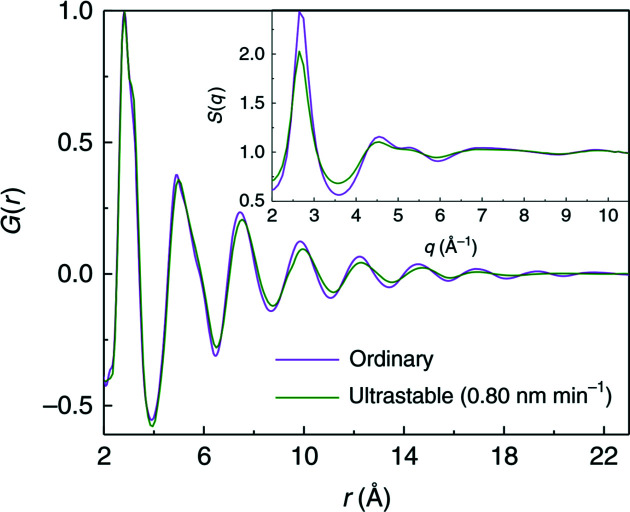
Structure factor *S*(*q*) (inset) and pair distribution function *G*(*r*) of 20 µm-thick ordinary metallic glass (produced by quenching) and a 2 µm-thick ultra-stable metallic glass produced by room-temperature chemical vapour deposition. The high quality of the (*r*) obtained from even such thin samples allows subtle differences in the local structure to be detected [from Luo *et al.* (2018 ▸)].

The detector characteristics, ideally suited to the needs of total scattering experiments, have allowed meaningful PDFs to be extracted from challenging samples such as 2 µm-thick metallic glass ribbons (Luo *et al.*, 2018 ▸), as shown in Fig. 15 ▸, microscopic samples within diamond anvil cells (Cerantola, 2020 ▸), as well as other embedded or dilute samples. The detailed performance of this detector for diffraction and total scattering experiments will be described in a subsequent article (Vaughan, 2020 ▸).

These characteristics, combined with the tomographic reconstruction algorithms described for XRD-CT applied to the scattering data from embedded amorphous phases within, for example, catalysts (Jacques *et al.*, 2013 ▸), batteries (Sottmann *et al.*, 2017 ▸) or even samples from cultural heritage (Jensen *et al.*, 2020 ▸), mean that high-quality *G*(*r*) can even be calculated from the reconstructed scattering patterns within the voxels inside these 3D objects. These *G*(*r*) thus calculated can be of sufficient quality for multiphase amorphous refinement (Sottmann *et al.*, 2017 ▸).

## 5. Conclusion and perspectives   

The upgrade of ID15A has produced an instrument optimized to perform *operando* studies on working systems using techniques such as XRD-CT/PDF-CT, high-speed and stroboscopic diffraction, and total scattering to reach unprecedented time and space resolution and sample quality from such techniques.

With further reduction in source size and increased flux following the ESRF-EBS upgrade, coupled with planned improvements in focusing optics, maps with ∼100 nm resolution, on a par or better than what can be achieved with direct absorption/phase-contrast tomography, are expected to be available. We can therefore envision in the coming years being able to deliver the same quality of four-dimensional data that XRD-CT makes available, on the length scale of the best direct three-dimensional data available from more classical tomographic methods. In order to exploit data with the highest spatial resolution, it may be essential to employ a mixed approach using both the XRD-CT methods currently applied and multigrain statistical methods (Sørensen *et al.*, 2012 ▸; Poulsen & Vaughan, 2019 ▸). Such an approach has already been demonstrated (Bonnin *et al.*, 2014 ▸). In order that X-ray dose rates do not rise in such a way as to cause a major increase in their effect on the reactions studied, it will be necessary to employ optimized fast-scanning strategies to limit exposure to that necessary to collect adequate data. In this sense, the total dose will remain comparable with present levels.

Furthermore, the first experiments using the technique of 6D tensor tomography (Malecki *et al.*, 2014 ▸; Gao *et al.*, 2019 ▸) have already been performed using high-energy WAXS (Grünewald, 2020 ▸) and we expect this technique, intrinsically time-consuming, to become more generally feasible following the ∼20× flux increase after the EBS upgrade.

In the field of PDF analysis, we expect to be able to perform experiments on amorphous systems with the time resolution currently only available for crystalline compounds. The unprecedented flux will also open up a large new range of samples for study by these methods, in particular the study of solvated molecules in order to study the field of solution chemistry, where the vast majority of reactions take place.

The flux increase will have a similarly significant effect on stroboscopic diffraction, where it will become feasible to use the storage-ring bunch clock and gating electronics, to allow a leap in time resolution to the picosecond domain. Whereas many stroboscobic experiments are expected to migrate to free-electron lasers in the near future, those requiring very high energy incident radiation will remain the domain of high-energy synchrotrons such as the ESRF.

## Figures and Tables

**Table 1:** Global beamline specifications and performance measured prior to the December 2018 shutdown, and anticipated at restart in 2020

	Pre-upgrade	EBS (if changed)
Photon source parameters (50 keV)		
RMS beam size (σ) (V × H)	4.4 µm × 49.6 µm	4.4 µm × 27.8 µm
Divergence (θ) (V × H)	4.5 µrad × 105.5 µrad	4.4 µrad × 6.8 µrad
Source 1	U22 undulator	CPMU18 undulator
Length	2 m	1.5 m
Period	22 mm	18 mm
Maximum deflection parameter (*K*)	1.788	1.75
Total power (6 mm gap)	6.9 kW	7.4 kW
Source 2		W76 Wiggler
Length		0.53 m
Period		76 mm
Maximum deflection parameter (*K*)		13.2
Total power		8.4 kW
Argon gas filter		
Length	1 m	
Pressure	350 mbar	
Metal-coated diamond filters		
Thickness (diamond)	300 µm	
Thickness (Cu)	0.8–25.8 µm	0.0–5.1 µm
Vertical focusing transfocator (TF1)		
Distance from source	32 m	
Distance from sample	33 m	
Lenses apex radius	100 µm	
Lenses vertical geometrical aperture	650 µm	
Focused vertical FWHM at sample	15 µm	15 µm
Double multilayer monochromator (DMLM)		
Materials	[Ni_93_V_7_/B_4_C]_500_	
*d*-spacing	1.996 nm	
Band-pass (Δ*E*/*E*)	0.37%	
Cooling	Water	
Energy range	20–70 keV	
Acceptance (20 keV)	4 mm (V) and 4.5 mm (H)	
Acceptance (95 keV)	4 mm (V) and 1.0 mm (H)	
Maximum flux density at sample (30 keV) (unfocused beam)	4.2 × 10^13^ photons s^−1^ mm^−2^	∼8.5 × 10^14^ photons s^−1^ mm^−2^
Laue–Laue monochromator		
Lattice	Si (111) or Si (311)	
*d*-spacing (111)	3.1347 Å	
*d*-spacing (311)	1.6370 Å	
Bending radius	30 m to ∞	
Band-pass (Δ*E*/*E*)	0.01–1%	
Cooling	Liquid N_2_	
Energy range (111)	40–180 keV	
Energy range (311)	80–250 keV	
Acceptance	2.0 mm (V) and 2.0 mm (H)	
Maximum unfocused beam at sample	1.2 mm × 6.4 mm (V × H)	
Maximum flux density at sample (70 keV) (unfocused beam)	4 × 10^12^ photons s^−1^ mm^−2^	∼1.3 × 10^14^ photons s^−1^ mm^−2^
Horizontal focusing transfocator (TF2)		
Distance from source	53 m	
Distance from sample	12 m	
Lenses radius	100 µm	
Lenses horizontal geometrical aperture	650 µm	
Focused vertical FWHM at sample	35 µm	18 µm
Microfocusing CRLs		
Distance from source	61–64 m	
Distance from sample	1–4 m	
Lenses radius	100 µm	
Lenses horizontal geometrical aperture	650 µm	
Vertical beam size at sample	0.5–2 µm	0.5–2 µm
Horizontal beam size at sample	3–9 µm	1.5–5 µm
Kirkpatrick–Baez focusing mirrors		
Distance from source	64.38 m (V) and 64.65 m (H)	
Distance from sample	0.62 m (V) and 0.35 m (H)	
Energy range	20–69 keV	
Output flux at 50 keV with DMLM	1.5 × 10^12^ photons s^−1^	4.5 × 10^13^ photons s^−1^
Acceptance	0.3 mm (V) and 0.3 mm (H)	
Beam size at sample (V × H)	0.3 µm × 0.6 µm	0.3 µm × 0.3 µm

**Table 2:** Operational parameters of the different experimental configurations used in EH3

	XRD-/PDF-/SAXS CT	Absorption-/phase-contrast CT	High-precision total scattering	High-frequency time-resolved
Energy (keV)	20–140	20–140	60–140	20–140
Primary optics	LLM/DMLM	LLM/DMLM/Pink	LLM/DMLM	LLM/DMLM
Secondary Optics	TF/CRL/KB	–	TF	TF/CRL/KB
Beam size				
Vertical (µm)	0.3–20	<6	100–200	15–200
Horizontal (µm)	0.3–50	<8	100–200	20–200
Spatial resolution				
Vertical (µm)	>0.3 µm	0.6/1.4/3.1	–	
Horizontal (µm)	>0.3 µm	0.6/1.4/3.1	–	
Detector	Pilatus/Maxipix	Imaging detectors	Pilatus	Pilatus/Maxipix
Goniometer	HR	HR	HR/HL	HR/HL

**Table 3:** Pilatus3 X CdTe 2M parameters compared with those of the Perkin Elmer XRD 1621

	Pilatus3 X CdTe 2M	Perkin Elmer XRD 1621
Detection technology	Hybrid photon counting	Flat panel
Sensor material	CdTe	CsI
Pixel size (µm)	172 × 172	200 × 200
Total number of pixels (H × V)	1475 × 1679	2024 × 2024
Maximum frame rate (Hz)	250 (500 with ROI)	15 (30 with 2 × 2 binning)
Point spread function (FWHM)	1 pixel	2 pixels
Energy threshold (keV)	8–40	None
Maximum count rate (photons s^−1^ pixel^−1^)	5 × 10^6^	Integrating detector
Non-linearity	<2% at 10^6^ counts s^−1^ pixel^−1^	
Counter depth	20 bit	16 bit
Dynamic range	20 bit	12.8 bit
Minimum exposure (ns)	200	3.3 × 10^7^
Image lag	0	∼1% after 100 ms
Readout time	0.95 ms	
File format	CBF, HDF5	Multiframe EDF

**Table 4:** Main characteristics of the X-ray detector systems with the PCO edge camera

	Low resolution		High resolution		
Objective magnification	1×	2×	5×	10×	20×
Pixel size (µm)	6.5 × 6.5	3.25 × 3.25	1.3 × 1.3	0.65 × 0.65	0.33 × 0.33
Field of view (mm)	16.6 × 14.0	8.3 × 7.0	3.3 × 2.8	1.7 × 1.4	0.83 × 0.70
Number of pixels	2560 × 2160 (H × V)				
Readout noise (r.m.s.)	1.5 e^−^				
Dark current	0.6 e^−^ pixel^−1^ s^−1^				
Minimum exposure time (ms)	0.5				
Maximum frame rate	100 Hz @ 2560 × 2180 pixels ROI				
	838 Hz @ 2560 × 256 pixels ROI				
File format	HDF5, multiframe EDF				

**Table 5:** Maxipix CdTe parameters

Detection technology	Hybrid photon counting
Sensor material	CdTe
Pixel size (µm)	55 × 55
Total number of pixels (H?V)	512 × 512
Maximum frame rate	350 Hz
Point spread function (FWHM)	1 pixel
Energy threshold	6–100 keV
Maximum count rate	2 × 10^5^ photons s^−1^ pixel^−1^
Counter depth	11810 counts
Dynamic range	1:11810
Minimum exposure	100 ns
Image lag	0
File format	HDF5, CBF, EDF

**Table 6:** Temperature control devices available at ID15A

Device	Temperature range (K)	Temperature resolution (K)	Sample size
Oxford 800plus cryostream	80–500	1	<2 mm
Linkam THMS-600 furnace	80–800	0.2	Capillary, film
Tomography furnace	300–1500	10	<10 mm
ESRF gas blower	300–1273	10	<3 mm

## References

[bb2] Ashiotis, G., Deschildre, A., Nawaz, Z., Wright, J. P., Karkoulis, D., Picca, F. E. & Kieffer, J. (2015). *J. Appl. Cryst.* **48**, 510–519. 10.1107/S1600576715004306 PMC4379438 25844080

[bb3] Beale, A. M., Jacques, S. D. M., Gibson, E. K. & Di Michiel, M. (2014). *Coord. Chem. Rev.* **277–278**, 208–223.

[bb4] Beyer, K. A., Zhao, H., Borkiewicz, O. J., Newton, M. A., Chupas, P. J. & Chapman, K. W. (2014). *J. Appl. Cryst.* **47**, 95–101.

[bb5] Biasci, J., Bouteille, J., Carmignani, N., Chavanne, J., Coulon, D., Dabin, Y., Ewald, F., Farvacque, L., Goirand, L., Hahn, M., Jacob, J., LeBec, G., Liuzzo, S., Nash, B., Pedroso-Marques, H., Perron, T., Plouviez, E., Raimondi, P., Revol, J., Scheidt, K. & Serrière, V. (2014). *Synchrotron Radiat. News* **27**(6), 8–12.

[bb6] Billinge, S. J. L. (2019). *Philos. Trans. R. Soc. A*, **377**, 20180413. 10.1098/rsta.2018.0413 PMC6501893 31030657

[bb7] Bleuet, P., Welcomme, E., Dooryhée, E., Susini, J., Hodeau, J.-L. & Walter, P. (2008). *Nat. Mater.* **7**, 468–472. 10.1038/nmat2168 18425135

[bb8] Bonnin, A., Wright, J. P., Tucoulou, R. & Palancher, H. (2014). *Appl. Phys. Lett.* **105**, 084103.

[bb9] Buslaps, T. (2020). In preparation.

[bb10] Cerantola, V. (2020). In preparation.

[bb11] Chavanne, J., Lebec, G., Penel, C., Revol, F., Kitegi, C., Garrett, R., Gentle, I., Nugent, K. & Wilkins, S. (2010). *AIP Conf. Proc.* **1234**, 25–28.

[bb12] Dabin, Y., Rostaing, G., Hignette, O., Rommeveaux, A. & Freund, A. K. (2002). *Proc. SPIE*, **4782**, 235.

[bb13] Di Michiel, M. (2020). In preparation.

[bb14] Friščić, T., Halasz, I., Beldon, P. J., Belenguer, A. M., Adams, F., Kimber, S. A. J., Honkimäki, V. & Dinnebier, R. E. (2012). *Nat. Chem.* **5**, 66–73. 10.1038/nchem.1505 23247180

[bb15] Gao, Z., Guizar-Sicairos, M., Lutz-Bueno, V., Schröter, A., Liebi, M., Rudin, M. & Georgiadis, M. (2019). *Acta Cryst.* A**75**, 223–238. 10.1107/S2053273318017394 PMC6396401 30821257

[bb16] Grünewald, T. (2020). In preparation.

[bb17] Halasz, I., Kimber, S. A. J., Beldon, P. J., Belenguer, A. M., Adams, F., Honkimäki, V., Nightingale, R. C., Dinnebier, R. E. & Friščić, T. (2013). *Nat. Protoc.* **8**, 1718–1729. 10.1038/nprot.2013.100 23949378

[bb18] Harding, G., Kosanetzky, J. & Neitzel, U. (1987). *Med. Phys.* **14**, 515–525. 10.1118/1.596063 3626990

[bb19] Honkimäki, V. (2020). In preparation.

[bb22] Jacques, S. D. M., Di Michiel, M., Beale, A. M., Sochi, T., O’Brien, M. G., Espinosa-Alonso, L., Weckhuysen, B. M. & Barnes, P. (2011). *Angew. Chem. Int. Ed.* **50**, 10148–10152. 10.1002/anie.201104604 21936040

[bb23] Jacques, S. D. M., Di Michiel, M., Kimber, S. A. J., Yang, X., Cernik, R. J., Beale, A. M. & Billinge, S. J. L. (2013). *Nat. Commun.* **4**, 2536. 10.1038/ncomms3536 24077398

[bb24] Jensen, K. M. Ø., Aluri, E. R., Perez, E. S., Vaughan, G. B. M., Di Michel, M., Schofield, E. J., Billinge, S. J. L. & Corr, S. *Nature Lett.* Submitted.

[bb25] Kieffer, J., Petitdemange, S. & Vincent, T. (2018). *J. Synchrotron Rad.* **25**, 612–617. 10.1107/S1600577518000607 29488943

[bb26] Kleuker, U., Suortti, P., Weyrich, W. & Spanne, P. (1998). *Phys. Med. Biol.* **43**, 2911–2923. 10.1088/0031-9155/43/10/017 9814526

[bb27] Liu, H., Kazemiabnavi, S., Grenier, A., Vaughan, G. B. M., Di Michiel, M., Polzin, B. J., Thornton, K., Chapman, K. W. & Chupas, P. J. (2019). *Appl. Mater. Interfaces*, **11**, 18386–18394. 10.1021/acsami.9b02173 31021598

[bb28] Liu, L., Rojac, T., Damjanovic, D., Di Michiel, M. & Daniels, J. (2018). *Nat. Commun.* **9**, 4928. 10.1038/s41467-018-07363-y PMC6250669 30467315

[bb29] Liuzzo, S. M., Carmignani, N., Chavanne, J., Farvacque, L., Perron, T., Raimondi, P. & White, S. (2018). *J. Phys. Conf. Ser.* **1067**, 032006.

[bb30] Luo, P., Cao, C. R., Zhu, F., Lv, Y. M., Liu, Y. H., Wen, P., Bai, H. Y., Vaughan, G. B. M., di Michiel, M., Ruta, B. & Wang, W. H. (2018). *Nat. Commun.* **9**, 1389. 10.1038/s41467-018-03656-4 PMC5895802 29643346

[bb31] Malecki, A., Potdevin, G., Biernath, T., Eggl, E., Willer, K., Lasser, T., Maisenbacher, J., Gibmeier, J., Wanner, A. & Pfeiffer, F. (2014). *Europhys. Lett.* **105**, 38002.

[bb33] Marion, P. & Zhang, L. (2004). *AIP Conf. Proc.* **705**, 320–323.

[bb32] Marion, P., Dabin, Y., Theveneau, P. & Zhang, L. (2002). *Second International Workshop on Mechanical Engineering Design of Synchrotron Radiation Equipment and Instumentation (MEDSI02)*, 5–6 September 2002, Argonne, IL, USA, pp. 433–442.

[bb34] Matras, D., Jacques, S. D. M., Poulston, S., Grosjean, N., Estruch Bosch, C., Rollins, B., Wright, J., Di Michiel, M., Vamvakeros, A., Cernik, R. J. & Beale, A. M. (2019). *J. Phys. Chem. C*, **123**, 1751–1760.

[bb35] Morawe, C. (2019). *AIP Conf. Proc.* **2054**, 060002.

[bb36] Morawe, C., Carau, D. & Peffen, J.-C. (2017). *Proc. SPIE*, **10386**, 1038603.

[bb37] Newton, M. A., Di Michiel, M., Kubacka, A. & Fernández-García, M. (2010). *J. Am. Chem. Soc.* **132**, 4540–4541. 10.1021/ja9107512 20225873

[bb38] Ponchut, C., Rigal, J. M., Clément, J., Papillon, E., Homs, A. & Petitdemange, S. (2011). *J. Instrum.* **6**, C01069.

[bb39] Poulsen, H. F. & Vaughan, G. B. M. (2019). *International Tables for Crystallography*, Volume H, edited by C. J. Gilmore, J. A. Kaduk & H. Schenk, pp. 601–616. International Union of Crystallography.

[bb49] Rossat, M., Vaughan, G. B. M., Snigirev, A., Wright, J. P., Bytchkov, A., Dabin, Y., Zhang, L., Garrett, R., Gentle, I., Nugent, K. & Wilkins, S. (2010). *AIP Conf. Proc.* **1234**, 744–747.

[bb50] Schultheiß, J., Liu, L., Kungl, H., Weber, M., Kodumudi Venkataraman, L., Checchia, S., Damjanovic, D., Daniels, J. E. & Koruza, J. (2018). *Acta Mater.* **157**, 355–363.

[bb40] Snigirev, A., Kohn, V., Snigireva, I. & Lengeler, B. (1996). *Nature*, **384**, 49–51.

[bb51] Snigirev, A., Snigireva, I., Vaughan, ... Rossat, M., Bytchkov, A. & Curfs, C. (2009). *J. Phys. Conf. Ser.* **186**, 012073.

[bb100] Sørensen, H. O., Schmidt, S., Wright, J. P., Vaughan, G. B. M., Techert, S., Garman, E. F., Oddershede, J., Davaasambuu, J., Paithankar, K. S., Gundlach, C. & Poulsen, H. F. (2012). *Z. Kristallogr.* **227**, 63–78.

[bb41] Sottmann, J., Di Michiel, M., Fjellvåg, H., Malavasi, L., Margadonna, S., Vajeeston, P., Vaughan, G. B. M. & Wragg, D. S. (2017). *Angew. Chem.* **129**, 11543–11547. 10.1002/anie.201704271 28650527

[bb42] Suortti, P. & Tschentscher, T. (1995). *Rev. Sci. Instrum.* **66**, 1798–1801.

[bb43] Sutter, J. P., Connolley, T., Drakopoulos, M., Hill, T. P. & Sharp, D. W. (2008). *Proc. SPIE*, **7077**, 70771N. 10.1107/S090904950802801X 18955764

[bb45] Tschentscher, T. (1996). *Rev. Sci. Instrum.* **67**, 3349.

[bb46] Vamvakeros, A., Jacques, S. D. M., Di Michiel, M., Matras, D., Middelkoop, V., Ismagilov, I. Z., Matus, E. V., Kuznetsov, V. V., Drnec, J., Senecal, P. & Beale, A. M. (2018). *Nat. Commun.* **9**, 4751. 10.1038/s41467-018-07046-8 PMC6232103 30420610

[bb48] Vaughan, G. B. M. (2020). In preparation.

[bb47] Vaughan, G. B. M., Wright, J. P., Bytchkov, A., Rossat, M., Gleyzolle, H., Snigireva, I. & Snigirev, A. (2011). *J. Synchrotron Rad.* **18**, 125–133. 10.1107/S0909049510044365 PMC3267637 21335897

